# Sirtuin-2 Regulates Sepsis Inflammation in *ob/ob* Mice

**DOI:** 10.1371/journal.pone.0160431

**Published:** 2016-08-08

**Authors:** Xianfeng Wang, Nancy L. Buechler, Ayana Martin, Jonathan Wells, Barbara Yoza, Charles E. McCall, Vidula Vachharajani

**Affiliations:** 1 Departments of Anesthesiology Wake Forest School of Medicine, Winston-Salem, NC, United States of America; 2 Department of Medicine Wake Forest School of Medicine, Winston-Salem, NC, United States of America; 3 Department of Surgery Wake Forest School of Medicine, Winston-Salem, NC, United States of America; Universidad Pablo de Olavide, SPAIN

## Abstract

**Objective:**

Obesity increases morbidity and resource utilization in sepsis patients. Sepsis transitions from early/hyper-inflammatory to late/hypo-inflammatory phase. Majority of sepsis-mortality occurs during the late sepsis; no therapies exist to treat late sepsis. In lean mice, we have shown that sirtuins (SIRTs) modulate this transition. Here, we investigated the role of sirtuins, especially the adipose-tissue abundant SIRT-2 on transition from early to late sepsis in obese with sepsis.

**Methods:**

Sepsis was induced using cecal ligation and puncture (CLP) in *ob/ob* mice. We measured microvascular inflammation in response to lipopolysaccharide/normal saline re-stimulation as a “second-hit” (marker of immune function) at different time points to track phases of sepsis in *ob/ob* mice. We determined SIRT-2 expression during different phases of sepsis. We studied the effect of SIRT-2 inhibition during the hypo-inflammatory phase on immune function and 7-day survival. We used a RAW264.7 (RAW) cell model of sepsis for mechanistic studies. We confirmed key findings in diet induced obese (DIO) mice with sepsis.

**Results:**

We observed that the *ob/ob*-septic mice showed an enhanced early inflammation and a persistent and prolonged hypo-inflammatory phase when compared to WT mice. Unlike WT mice that showed increased SIRT1 expression, we found that SIRT2 levels were increased in *ob/ob* mice during hypo-inflammation. SIRT-2 inhibition in *ob/ob* mice during the hypo-inflammatory phase of sepsis reversed the repressed microvascular inflammation *in vivo* via activation of endothelial cells and circulating leukocytes and significantly improved survival. We confirmed the key finding of the role of SIRT2 during hypo-inflammatory phase of sepsis in this project in DIO-sepsis mice. Mechanistically, in the sepsis cell model, SIRT-2 expression modulated inflammatory response by deacetylation of NFκBp65.

**Conclusion:**

SIRT-2 regulates microvascular inflammation in obese mice with sepsis and may provide a novel treatment target for obesity with sepsis.

## Introduction

Sepsis and septic shock are the leading causes of death in non-coronary intensive care units. In the United States alone, they kills more than 200,000 patients each year, with an annual cost of treatment $16 billion[1]. The acute systemic inflammatory response of sepsis quickly transitions from an early/hyper-inflammatory phase to a late/hypo-inflammatory phase with persistent multi-organ dysfunction [2,3]. Evidence suggests that the hypo-inflammatory phase of sepsis, the host is unable to clear the pre-existing infection or superadded infections effectively and succumb to sepsis[4]. Immune-repressor hypo-inflammatory sepsis phenotype switches from glycolysis to fatty acid metabolism as a primary source for ATP production [5]. While some patients die early during sepsis, most sepsis-related deaths occur during the late/hypo-inflammatory phase of sepsis [6]. Over 30 different treatment modalities, all targeting early sepsis have failed to improve survival [7]. Evidence supports a new approach of treating sepsis by enhancing the repressed immune response[4].

The repressive stage of sepsis is characterized by the phenomenon of “endotoxin tolerance”, first described by Beeson as a decreased response to subsequent endotoxin stimulation after the first typhoid vaccine in rabbits [8]. Repressed immunity/endotoxin tolerance occurs after sepsis initiation, thereby providing a biomarker of immune disruption; endotoxin tolerance can be used as a test for “immune function” in mouse. Using endotoxin (E. coli lipopolysaccharide: LPS) as a “second hit” to study the response to further inflammatory stimuli, we can test whether or not a cell/organism immunity is intact (response to second-hit preserved) vs. repressed (response to second-hit impaired). We have shown that in lean WT mice with sepsis, the initial hyper-inflammatory (endotoxin responsive) phase transitions to hypo-inflammatory/repressed immunity (endotoxin tolerant) phase[2].

Obesity, a disease of rising prevalence, affects more than a third of the adult USA population [9]. Healthcare-related expenditure in obese individuals is projected increase by $549 billion by the year 2030[10] if the obesity trend continues. Although the association between sepsis- mortality and obesity is debated, literature, unequivocally, shows increased morbidity and resource utilization in obese with critical illness [11,12].

Mounting evidence supports a role for the sirtuin family of highly conserved NAD+ dependent deacetylases in directing the course of sepsis [5,13,14], but whether and how this concept applies to obesity is unknown. First identified in yeast, the seven-member mammalian sirtuins (SIRT-1-7) are now recognized as regulators of metabolic homeostasis and guardians of metabolism, immunity and bioenergetics[13] [15]. We discovered that in cell and lean mouse models of sepsis that nuclear SIRT-1 couples with nuclear NFκB p65/ RelB, nuclear SIRT-6 and mitochondrial SIRT-3 as homeostasis checkpoints during acute sepsis-inflammation [5,14]. Sepsis shifts metabolism between the early activated and subsequent repressive stage by switching glycolysis as an energy source to mitochondrial oxidation of fatty acids as primary energy. How sirtuins affect obesity with inflammation is not clear, but obesity alone can decrease sirtuin expression [16].

Microvasculature, with its highly strategic interface between systemic circulation and local tissue environment, plays a critical role in modulating the course of sepsis inflammation and organ function [2]. Interactions between circulating immune cells and endothelial cells in the microcirculation are rate- determining factors for inflammation [17]. This critical area of inflammation/ immune regulation is often missing from the studies of inflammatory and immune diseases, but when performed *in vivo* offers an insight not available from *ex vivo* or *in vitro* cell or tissue research. We have adapted this tool to study microvascular inflammation of sepsis temporally and to assess immune function by studying leukocyte adhesion *in vivo*, in response to endotoxin as a second-hit at different time points post-sepsis.

In this project, we determined in *ob/ob* mice: 1) the effect of obesity on the repressed inflammation phenotype (hypo-inflammation) and its duration; 2) whether sirtuins contribute to the nature and duration of sepsis reprogramming of inflammation and immunity; 3) if modifying sirtuin function alters inflammation and immune sepsis reprogramming; and 4) the effect of blockage of sirtuins on duration of these phases and sepsis-survival. Unlike WT mice, we show that in *ob/ob* mice with sepsis, the initial phase of hyper-inflammation is exaggerated and shortened and the repressed/ hypo-inflammatory response is prolonged. In a distinct contrast our reports in lean septic mice [2], we implicate SIRT-2 deacetylase and not SIRT-1 as a regulator of microvascular function and survival in *ob/ob* mice. In our sepsis cell model, we show that SIRT-2 deacetylates master inflammatory and immune transcription factor NFkB p65, which is known to repress transcription of inflammation and immune activator genes, like TNF-α, IL-6 etc. [18]. Lastly, we examined the key findings in our manuscript in mice with diet induced obesity (DIO) with sepsis.

## Methods

Animals: Study was approved by the Institutional Animal Care and Use Committee of the Wake Forest School of Medicine and experiments performed according to the NIH guidelines. The wild type (WT: C57Bl/6; 6–8 week old) and *ob/ob* (B6.Cg-Lepob/J; 6–8 week old) and diet induced obesity (DIO; 13–15 week old) mice were purchased from Jackson Laboratories (Bar Harbor, ME, USA). Diet induced obesity was induced in C57Bl/6 mice who received 60% fat diet (Research Diets Inc. cat no: D12492) for 7–9 weeks, starting as 6 weeks of age. A group of mice were injected with a single dose of either EX-527 (10mg/kg) or AK-7 40mg/kg (4ml/kg) or equivalent volume of DMSO (Vehicle) (4ml/kg) intraperitoneally, 18 hours post-sepsis. These doses of EX-527 and AK-7 in mice were per literature [2] [19].

Cecal ligation and puncture (CLP): Mice were anesthetized using isoflurane anesthesia (1–3% Isoflurane/ O2 mixture via nose cone). CLP was used to induce sepsis as described in the literature [2]. Laparotomy was performed; cecum identified, ligated, perforated two times with a 22-guage or a 25-gauge needle (see below), contents returned, abdomen closed in two layers (peritoneum and skin) and animal received 1 ml normal saline fluid resuscitation subcutaneously. Sham-operated mice underwent laparotomy and fluid resuscitation without cecal ligation and puncture.

*Sepsis “dose titration in ob/ob” mice*: We started out studying model of CLP (sepsis) used in previous publications [2,20] and observed that the *ob/ob* mice had significantly decreased survival compared to WT lean mice. As shown in S1A Fig, we observed that using 22-guage needle and two punctures model of sepsis (CLP 22.2), there was 0% 7-day survival in *ob/ob* mice vs. 40% in WT mice; all *ob/ob* mice died within 48 hours. As shown in S1B Fig, with CLP using 25-guage needle and two punctures model of sepsis (CLP 25.2) we were able to achieve a comparable (30% survival) in *ob/ob* mice with our previous publications[2,20]) and decided that CLP25.2 was the optimal dose of sepsis in *ob/ob* mice to study early and late sepsis. However, the survival in WT mice with CLP25.2 was 100% (S1B Fig). Mice with CLP25.2 (Sepsis)/ Sham surgeries were used for tissue harvest or intravital fluorescent video microscopy at specified time points post-surgery as indicated.

*Endotoxin tolerance in vivo*: We used *in vivo* endotoxin tolerance to test immune function in sepsis mice at different time points as indicated. To do this, mice received either E. coli lipopolysaccharide O111:B4 (LPS: 5μg/mouse intraperitoneally: 4 hours of stimulation) or normal saline (NS) as a “second hit” after CLP. We used CLP25.2 in WT and obese mice.

*Pain and distress*: All mice were monitored at least twice per day. Pain and distress were relieved using buprenorphine analgesia (0.05mg/kg body weight; intraperitoneally). Pain scoring system, indications for analgesia and humane end points for euthanasia are described in in detail in Table 1. We used isoflurane overdose followed by cervical dislocation (secondary method) for euthanasia. Despite rigorous monitoring, the rate of unexpected deaths was 15–20% and cause of death was found to be due to intra-abdominal abscess formation.

**Table 1:** Monitoring pain and distress.

Pain score	Observation	Recommendation
**0**	Asleep or awake. Normal appearance and behavior. Respiratory rate normal	No analgesic indicated
**1**	Mild agitation. Depressed and uninterested in surrounding, frequent position change of reluctant to move. Mild changes in appearance, eyes partially closed, decreased interaction. Respiratory rate up to 30% above normal	Buprenorphine 0.05mg/kg intraperitoneally. Frequent (at least every four hours) monitoring. Euthanasia if persistent score 1 for four hours or progression to score 2.
**2**	Moderate agitation, restless and uncomfortable. Moderate changes in eyes, sunken or glazed, unthrifty. Moderate changes in behavior, less mobile, less alert, unaware of surroundings. Reluctant to move, but will if coaxed. Respiratory rate 30–45% above normal.	Buprenorphine 0.05mg/kg intraperitoneally. Monitoring at least 2 hours post-analgesia. If unchanged, euthanasia.
**3**	Extremely agitated thrashing. Severe changes in appearance. Eyes pale, enlarged pupils. Guarding, hunched in appearance, legs in abnormal position, teeth grinding. Respiratory rate more than 45% above normal.	Euthanasia.

Intravital fluorescent video microscopy (IVM): Mice were anesthetized using ketamine (150mg/kg) + Xylazine (7.5 mg/kg) intramuscularly. Intravital microscopy procedures were described previously [2]. The mice underwent carotid artery (to measure invasive blood pressure, MAP monitoring) and jugular venous cannulations (to inject platelets/Rhodamine G intravenously), laparotomy incision opened, small intestine exteriorized and the small intestinal microcirculation was studied (n = 4–6 mice/group) [2]. *In vivo* visualization of leukocyte achieved by injecting mice with Rhodamine G (labeled red) while platelets were labeled green *ex vivo* with carboxyfluorescein diacetate succinimidyl ester (CFSE: Sigma-Aldrich; St Louis, MO, USA; 90 μM) to allow simultaneous monitoring of leukocytes and platelets.

The details of the platelet isolation technique are as outlined previously [20,21]. The platelets (n = 100 x 10^6^) were infused intravenously over 5 min (yielding <5% of the total platelet count) and allowed to circulate for a period of 5 min before recording on a DVD. Literature suggests that these platelet isolation procedures have no significant effect on the activity or viability of isolated platelets [22].

The post-capillary venules (n = 3–5/mouse; 4–6 mice per group) were recorded (1 min 20 seconds each) and leukocyte/ platelet adhesion quantified. Cell (leukocyte/platelet) was considered adherent if stationary for at least 30 consecutive seconds of the one minute recording. The mean of the average values of leukocyte adhesion determined in each mouse were then used to generate a group mean.

Immunohistochemistry of small intestinal tissue: Small intestinal tissue was harvested and fixed frozen sections of tissue were stained using antibodies against SIRT-2(Santa Cruz Biotechnology, Inc. Santa Cruz, CA, USA), E-selectin and ICAM-1 (BD biosciences, San Jose, CA, USA) and Von Willebrand factor (VWF, Abcam, Inc. Cambridge, MA, USA). Cy^™^3-conjugated labeled secondary antibodies for SIRT-2, E-selectin, ICAM-1 and FITC conjugated secondary antibody for VWF were purchased from Jackson Immuno Research Laboratories, Inc. (West Grove, PA, USA) [2,20]. Virtual images were captured as described previously[2] and immunofluorescence quantification were performed using NIH Image J software [23].

Cell Culture: RAW264.7 (ATCC^®^ TIB-71^™^: RAW) and HEK 293 (ATCC^®^ CRL- 1573^™^: HEK) cells were obtained from ATCC. Cells were cultured in Dulbecco’s Modified Eagle’s Medium (DMEM) containing 10% heat-inactivated fetal bovine serum (iFBS), 100units/ml penicillin, and 100 mg/ml streptomycin at 37°C and 5%CO2. Early passage (2–10) cultures were used in all experiments.

SIRT2 overexpression and knockdown: To overexpress SIRT2 protein, SIRT-2 (or control plasmid pcDNA3) plasmids were co-transfected with p65 or/and CBP as indicated based on reported in literature [24]. Plasmids were diluted in Opti-MEM medium, followed by the addition of SuperFect (QIAGEN, #301305). The transfection complex was added to HEK 293 cells and the medium was replaced with DMEM containing 10% iFBS and whole cell lysates were collected for western blotting 48 hours post-transfection. HEK cells were used for overexpression experiment due to low transfection efficiency for SIRT2 plasmid in RAW cells.

For knock-down of SIRT-2, we co-transfected RAW cells with p65 and CBP plasmids together with either SIRT2 siRNA (si-SIRT2) (Dharmacon Cat #: L-061727-02-0005) or scrambled control (si-Ctrl) (Dharmacon Cat #: D-001810-10-05) with RNAiMax reagent and incubated and RAW cells were detached and suspended DMEM containing 10% iFBS. The whole cell lysates were collected for western blotting.

We obtained pcDNA3-β-FLAG-CBP-HA (Addgene plasmid # 32908), SIRT2 Flag (Addgene plasmid # 13813) and pCMV4 p65 (Addgene plasmid # 21966) from Addgene.

RNA extraction and RT-PCR: RNA extraction method and RT-PCR were completed as described previously [2,20]. The mRNA expression was quantified by quantitative real-time PCR with SensiFAST Probe Lo-ROX One-Step Kit (Bioline, BIO-78005). GAPDH was used to normalize the gene expression data. Relative quantification was calculated using the ΔΔ comparative threshold formula. All samples were run in quadruplicates to calculate average and SE value. TaqMan primer/probes were purchased from Invitrogen (Grand Island, NY).

Western blot analysis: Western blot was completed as previously described[20], using antibodies against GAPDH or cyclophilin A (CPA) or GAPDH as loading control. The membrane was incubated with antibodies against SIRT-2 (Santa Cruz Biotechnology, sc-20966), Ac-p65 (Cell Signaling Technology, #3045S), total p65 (anta Cruz Biotechnology, sc-372), GAPDH (Invitrogen, #AM4300) or CPA (Millipore, #07313) overnight followed by the incubation with Alexa Fluor 680-conjugated secondary antibodies (827–10080 or 827–10081; LI-COR Biosciences, Lincoln, NE) and signal was developed with a Li-COR Odyssey Infrared Imager system (Li-COR Biosciences). Molecular weight of SIRT2 (39 or 43 kD: two isoforms), is very close to molecular weight of GAPDH (42kD). So we chose Cylophilin A (18kD) as loading control sometimes, as used in literature [25] (https://www.bio-rad-antibodies.com/western-blot-loading-controls-antibodies.html).

Whole blood leukocytes: Blood was collected via carotid arterial cannulation in anesthetized mice, peripheral leukocytes were stained with anti-CD45 (eBioscience, Inc., San Diego, CA, USA) and anti-PSGL-1 (BD Biosciences, San Jose, CA, USA) antibodies. The samples were assayed by flow cytometry using a BD Acuri C6 flow cytometer (BD Biosciences, San Jose, CA, USA) as described before[2]. We gated for CD45+ cells (total leukocytes). PSGL-1 expression was determined by MFI analysis of leukocytes (PSGL-1+/CD45+ cells). MFI values were normalized to control.

Statistics: All data were analyzed using Graph Pad Prism 6.0 (Graph Pad Software, La Jolla, CA, USA). Analyses with more than three groups were analyzed using one-way ANOVA or two-way ANOVA with Tukey’s post hoc comparisons as appropriate. A p<0.05 was designated as significant. Leukocyte adhesion was analyzed using Tukey-Kramer post-hoc analysis (Statview, SAS; Cary, NC, USA). A p<0.05 was designated as significant. Log rank test was used to compare survival between the groups in the Kaplan-Meier survival curves and a p < 0.05 was designated as significant.

## Results

### *Ob/ob* mice rapidly enter a prolonged immune-repressor hypo-inflammatory phase of sepsis

**Fig 1:**
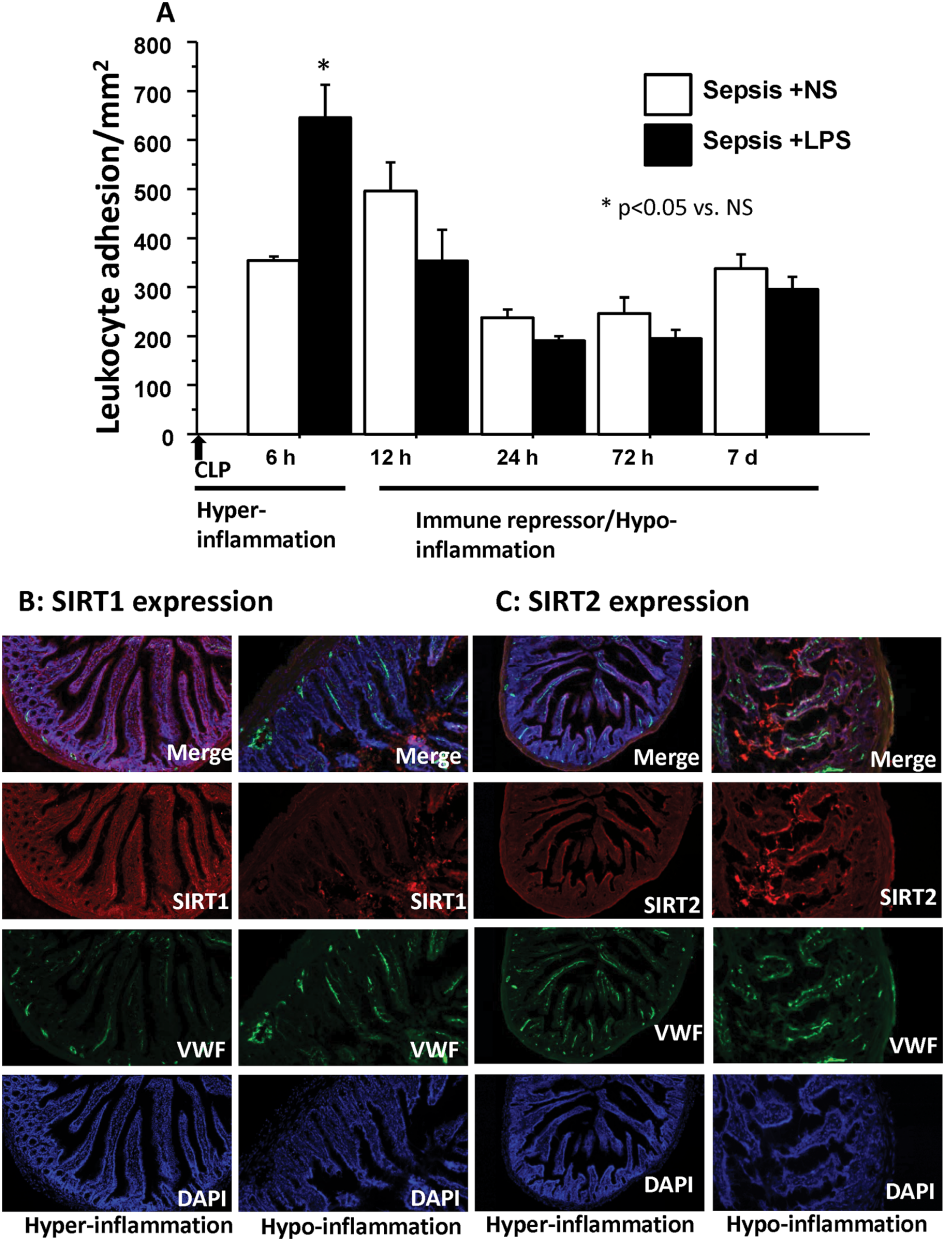
*Ob/ob* mice rapidly and persistently enter the sepsis repressor hypo-inflammatory phase. A: *Ob/ob* mice show a prolonged repressor/ hypo-inflammatory phase of sepsis: In *ob/ob* mice with sepsis, leukocyte adhesion in small intestinal microcirculation (mice n = 5/group) peaked at 12h in Vehicle (normal saline) group and decreased in after 18 h in Sepsis (CLP) group. LPS challenge in sepsis mice significantly increased leukocyte further (vs. Vehicle) only in 6h Sepsis group (hyper-inflammatory) while it did not in 12, 24, 72h and 7 day Sepsis groups, indicating endotoxin tolerance (immune repression/hypo-inflammation). n = 4–6/group * p<0.05 vs. respective Vehicle group Tukey‘s post-hoc analysis; error bars: s.e.m. B and C: Immunohistochemistry of small intestinal tissue were stained for SIRT-1 (Cy3: red; 1B) SIRT-2 (Cy3: red; 1C) during the hyper- and hypo-inflammatory phases of sepsis. SIRT-2 (Cy3: red), Von Willebrand factor (VWF, FITC: green), nuclear stain (DAPI: blue) and merged color image show that SIRT-1 expression decreased while SIRT-2 expression increased during immune repressor/ hypo-inflammatory phase (24 h) Sepsis compared to hyper-inflammatory phase (6 h).

We defined temporal changes in leukocyte adhesion, using endotoxin stimulation as a “second hit” to study microvascular response to inflammatory stimulus at different time points after sepsis-induction. Increased leukocyte adhesion in response to endotoxin (endotoxin-responsiveness) indicates immune-competence while endotoxin tolerance serves as a marker for putative immune-repression *in vivo*. To achieve this, we challenged WT and *ob/ob*- septic mice with either normal saline (NS) or lipopolysaccharide (LPS: endotoxin) as a second-hit at different time points after sepsis and studied small intestinal microcirculation for leukocyte adhesion. The leukocyte adhesion in Sepsis +NS in *ob/ob* mice peaks at 12h post-sepsis and decreases thereafter (Fig 1A). The leukocyte adhesion in WT mice with CLP25.2 peaked at 6 hours and decreased thereafter (supportive information S1 Fig). The leukocyte adhesion in Sepsis+ NS groups remained significantly higher in *ob/ob* vs. WT mice in 6, 12, 24 and 72 hours post-CLP (data not shown).

In *ob/ob* mice, when challenged with endotoxin (Sepsis +LPS), further accentuation of the leukocyte adhesion (vs. Sepsis +NS) occurred only in the first 6h after sepsis to be followed by an endotoxin-tolerant phase; the endotoxin tolerance (repressor/hypo-inflammatory phase) persists for up to 7 days in septic *ob/ob* mice (Fig 1A). This suggests a prolonged hypo-inflammation/ delayed sepsis resolution in *ob/ob* mice. As shown in Table 2, there were no significant differences in body weight and carotid arterial invasive mean arterial blood pressure (MAP) between the groups. In WT counterparts with CLP 25.2, Sepsis+ LPS groups had significantly increased leukocyte adhesion vs. Sepsis+NS at all the time points studied, i. e. 6, 12, 24 and 72 hours post-sepsis. This suggests that microvasculature remained endotoxin-responsive (S2A Fig). Thus, we show while WT mice remain endotoxin responsive with 100% survival, the *ob/ob* mice show prolonged endotoxin tolerance with this model of cecal ligation and puncture (CLP 25.2).

**Table 2:** Weight in grams and mean arterial blood pressure (MAP) in different groups.

	Body Weight (gms) Mean± SEM		MAP (mmHg) Mean± SEM	
	NS	LPS	NS	LPS
6 h sepsis	41.83±3.9	40.97±4.3	55.6±1.8	61.20±3.2
12 h sepsis	36.76±0.53	37.85±1.4	56.0±4.6	64.75±5.6
24 h sepsis	35.56±0.33	36.92±1.5	60.66±0.33	58.33±8.6
72 h sepsis	35.02±1.3	35.27±1.05	62±2.9	68.20±6.61
7 days	37.01±1.1	35.57±0.80	69±5.3	72.4±5.8

Body weight and carotid arterial mean arterial blood pressure in different groups of *ob/ob* mice with and without LPS second-hit studied show no significant differences from each other. Similarly, the mean arterial blood pressures (MAP) between different groups were not significantly different from each other.

### SIRT-2 expression in *ob/ob* septic mice increases during immune repressor hypo-inflammatory phase

We first studied whether there was a difference in SIRT-1 expression in small intestinal tissue during hyper-inflammatory phase (6 hours) vs. hypo-inflammatory phase (24 hours) of sepsis. We showed that in *ob/ob* mice, SIRT-1 expression during hypo-inflammatory phase of sepsis decreased vs. hyper-inflammatory phase as shown in Fig 1B. Next, we investigated whether there was change in SIRT-2 expression and found that SIRT-2 expression in the small intestinal tissue of *ob/ob* mice increased during the hypo-inflammatory phase vs. hyper-inflammatory phase as shown in Fig 1C. We found similar results, i. e. decreased SIRT-1 and increased SIRT2 expression during the hypo-inflammatory phase in the liver tissue of *ob/ob* mice with sepsis (not shown). We also studied the SIRT2 expression in WT mice with sepsis during corresponding time points (6 and 24 hours post-sepsis) and show in supportive information S2B Fig that in WT mice, the SIRT2 expression remained unchanged at 6 vs. 24 hours post-sepsis. Taken together, we show that the *ob/ob* mice show prolonged endotoxin tolerance with increased SIRT2 expression. Thus, SIRT-2 and not SIRT-1 expression was increased during hypo-inflammatory phase of *ob/ob*-sepsis mice.

### SIRT-2 and not SIRT-1 inhibition during sepsis repressor/ hypo-inflammatory phase improves sepsis survival in *ob/ob* mice

**Fig 2:**
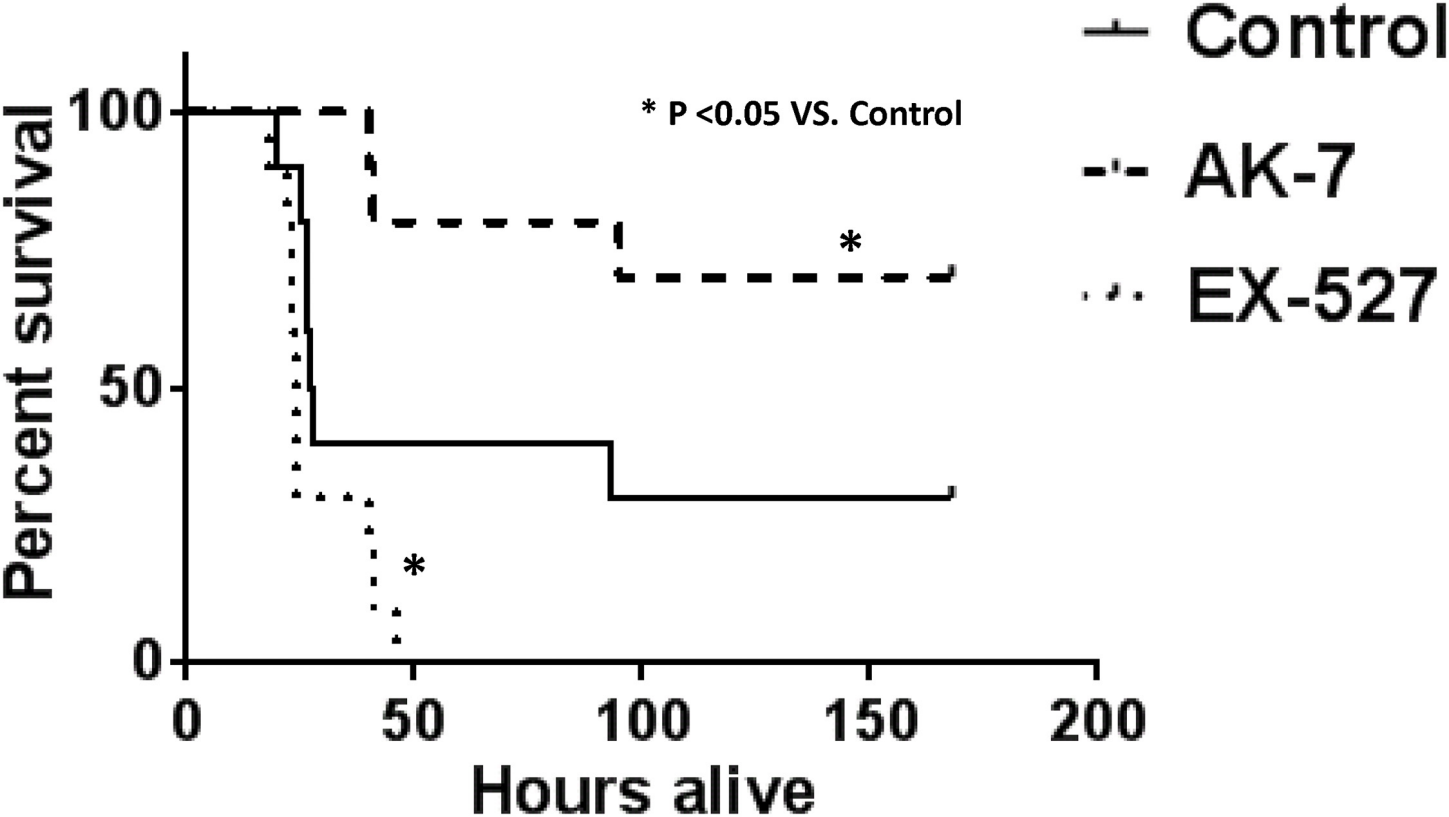
SIRT-2 inhibition during sepsis repressor/ hypo-inflammatory phase improves survival. We examined the effect of SIRT-1 (EX-527) and SIRT-2 (AK-7) inhibitors vs. vehicle treatment during the hypo-inflammatory phase of sepsis on 7-day survival in *ob/ob* mice. As shown in Fig 2, there was a significant decrease in 7-day survival of mice treated with EX-527 (EX-527: 0% vs. Vehicle: 30%). However, there was a significant increase in survival of *ob/ob* septic mice treated with AK-7 during the hypo-inflammatory phase of sepsis (AK-7: 70%vs. Vehicle: 30%).* p<0.05 vs. Sepsis + Vehicle using Log-Rank test.

To further investigate the potential differences in *ob/ob* vs. WT sepsis, we tested whether treatment with SIRT1-specific inhibitor EX-527 improved 7- day survival in *ob/ob* mice. In contrast to our findings in lean/WT sepsis, where EX-527 improved survival, [2], we found that in *ob/ob* mice with sepsis EX-527 significantly decreased survival in *ob/ob* mice with sepsis. (Fig 2). Since SIRT-2 expression appears to be increased in *ob/ob* mice with CLP25.2, we tested the effects of SIRT-2 specific inhibitor AK-7 on 7-day sepsis survival and observed there was a significant increase in survival of *ob/ob* septic mice treated with AK-7 (Fig 2). Thus, the data suggest that SIRT-2 and not SIRT-1 plays a crucial role in *ob/ob* mice with sepsis.

### The role of SIRT-2 in the endotoxin tolerant RAW cells

**Fig 3:**
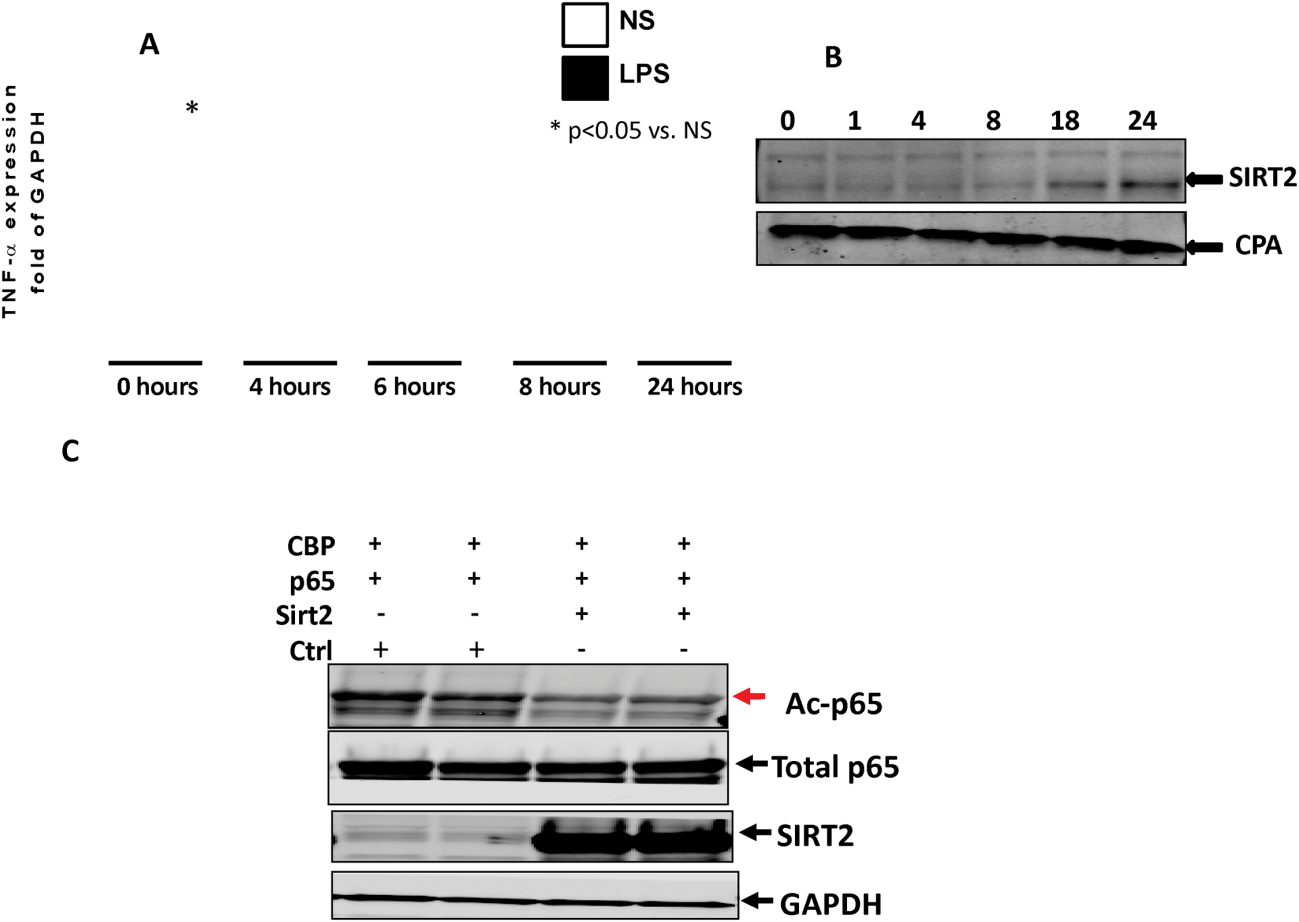
Role of SIRT-2 in the endotoxin tolerant RAW cells. A: RAW cells undergo endotoxin tolerance as early as 4h after LPS stimulation: To study endotoxin tolerance in RAW cells, we stimulated cells with LPS (100ng/ml) and re-stimulated with another LPS challenge (100ng/ml) at 0, 4, 6, 8, and 24 hour time points for four hour. We studied TNF-α mRNA expression. The cells increased TNF-α mRNA expression in response to LPS re-stimulation only at 0h time point. RAW cells were unable to increase TNF-α mRNA further to LPS re-stimulation at 4, 6, 8 and 24h time points, indicating endotoxin tolerance. * p<0.05 vs. respective NS group Tukey‘s post-hoc analysis; error bars: s.e.m. B: SIRT-2 protein expression increased during endotoxin tolerant phase in RAW cells: RAW cells were treated with LPS (100ng/ml) for 0, 1, 4, 8, 18, and 24 h. Whole cell lysates were collected for western blotting of proteins SIRT-2 and CPA (housekeeping gene). Representative image out of three experiments shows that was increased in SIRT-2 expression in 18 and 24h after LPS stimulation. C SIRT-2 deacetylates NFkB p65: We studied the effect of SIRT-2 expression on NFkB p65 acetylation using HEK293 cells. SIRT-2 plasmid was co-transfected with p65 or/and CBP plasmids into HEK293 cells (to increase baseline p65 acetylation) and blotted for antibodies against Ac-p65, total p65, SIRT-2 and GAPDH. NFkB p65 acetylation (Ac-p65) increased in cells with transfection with p65+CBP while it decreased in cells transfected with p65+CBP+SIRT-2, indicating SIRT-2 directly deacetylates NFkB p65.

We next sought to elucidate the mechanism role of SIRT-2 in endotoxin tolerance as it relates to SIRT-2 expression, using mouse macrophage cell line, the RAW cells. Endotoxin stimulation and subsequent tolerance is described in RAW cells has been studied as a cell culture sepsis model in the literature [26]. We primed RAW cells with LPS 100 ng/ml for indicated times (0h, 4h, 6h, 8h, and 24h) and then re-stimulated with LPS/ vehicle (normal saline: NS) as a second-hit for additional 4h and then assessed TNF-α mRNA expression and show that the second dose of LPS is unable to further increase TNF-α mRNA expression, confirming previous studies and further showing endotoxin tolerance in RAW cells as early as 4h after the first dose of LPS (Fig 3A). We next examined SIRT-2 expression in endotoxin tolerant RAW cells. Similar to *ob/ob* tissue (Fig 1), we show increased SIRT-2 expression in endotoxin tolerant RAW cells (Fig 3B).

Sirtuins directly deacetylate and inactivate NFkB p65 [18]. Next, we studied whether SIRT-2 deacetylates NFκBp65. Due to very low plasmid transfection efficiency in RAW cells, we studied p65 deacetylation as a proof of principle study, first in HEK cells. We studied acetylated (AC-p65) expression in cells transfected with SIRT-2 vs. empty (Ctrl) plasmid with p65+CBP (to increase baseline p65 acetylation) co-transfection. We then studied AC p65 using western blot analysis. As shown in Fig 3C, AC-p65 expression decreased significantly in cells transfected with p65+CBP+SIRT-2 compared to those with Ctrl+ p65+CBP. Taken together, our data suggest that in a cell model that SIRT-2 participates in endotoxin tolerance by deacetylating and inactivating NFkB p65.

### SIRT-2 inhibition reverses endotoxin tolerance in RAW cells

**Fig 4:**
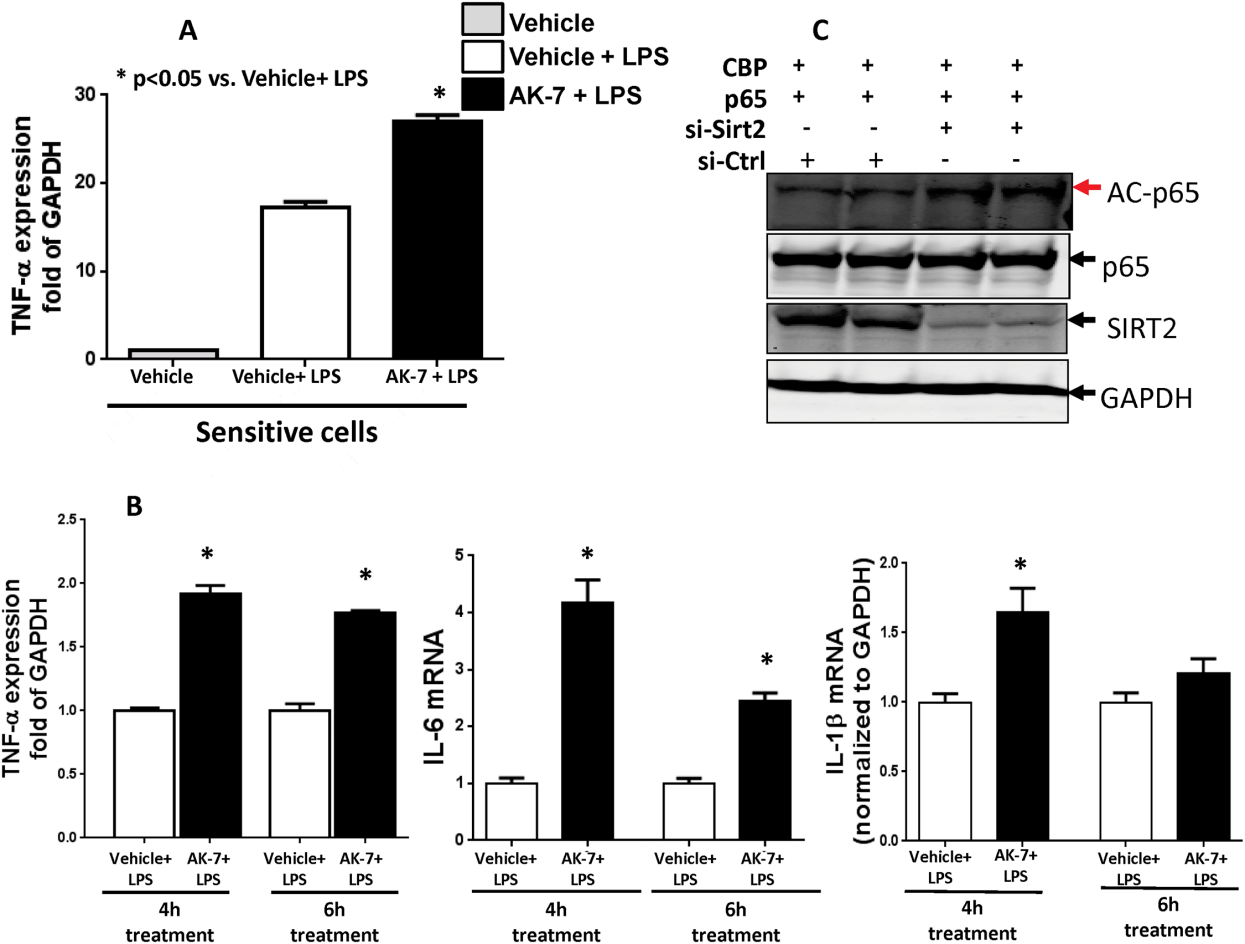
SIRT-2 inhibition reverses endotoxin tolerance in RAW cells. A: SIRT-2 inhibitor AK-7 enhanced LPS induced cytokine response in sensitive cells. RAW cells were pre-treated with vehicle (DMSO) or AK-7 (25uM) for 1h before the stimulation with normal saline or LPS (100ng/ml) for 4h and TNF-α mRNA expression levels were studied in different groups. There was significant increase in TNF-α mRNA in AK-7 +LPS vs. Vehicle +LPS group. * p<0.05 vs. Vehicle +LPS using Tukey‘s post-hoc analysis; error bars: s.e.m. B SIRT-2 inhibitor AK-7 reverses endotoxin tolerance in RAW cells: Endotoxin tolerance was induced with LPS (100ng/ml) treatment for up to 24h. AK-7 (25uM) was added to cell culture at 4h and 6h after the beginning of LPS stimulation, followed by further stimulation with Vehicle or LPS (100ng/ml) for 4h and TNF-α, IL-6 and IL-1β mRNA expression in different groups were studied. While Vehicle treated groups remained endotoxin tolerant, there was a significant increase in all three cytokine mRNA expression in AK-7 +LPS vs. Vehicle +LPS treated groups. * p<0.05 vs. Vehicle +LPS using Tukey‘s post-hoc analysis; error bars: s.e.m. C: SIRT-2 deficiency increases NFkB p65 acetylation: We studied the effect of SIRT-2 deficiency on NFkB p65 acetylation using RAW cells. We treated RAW cells with SIRT-2 siRNA (si-SIRT2) or scramble control (si-Ctrl), and co-transfected with p65 and CBP plasmids (to increase baseline p65 acetylation) and blotted for antibodies against Ac-p65, total p65, SIRT-2 and GAPDH. NFkB p65 acetylation (Ac-p65) increased in cells with transfection with p65 + CBP. Cells treated with p65+CBP+si-SIRT-2 showed further increase in AC-p65 vs. p65+CBP+si-Ctrl, indicating SIRT-2 directly deacetylates NFkB p65.

First we studied the effect of SIRT-2 inhibitor AK-7 pre-treatment on hyper-inflammatory phase in the RAW cells. We show that there was a significant increase in TNF-α mRNA expression in endotoxin sensitive RAW cells pre-treated with AK-7 vs. Vehicle (Fig 4A). Next, we determined whether SIRT-2 specific inhibitor AK-7 could reverse endotoxin tolerance. We treated cells at 4 and 6h post-LPS (when cells are transitioning to the repressed state) with either vehicle or AK-7 and then re-stimulated with LPS. We show that AK-7 treated cells significantly increase TNF-α, IL-6 and IL1-1β mRNA levels (Fig 4B) compared to Vehicle treated cells, suggesting that SIRT-2 inhibition can reverse endotoxin tolerance in RAW cells.

To further study the effect of SIRT-2 deficiency on AC-p65 expression, we treated RAW cells with SIRT2 siRNA (si-SIRT2) vs. scramble siRNA (si-Ctrl) co-transfected with p65+CBP. We then studied AC-p65 expression in these cells using western blot analysis shown in Fig 4C. AC-p65 increased further in RAW cells treated with si-SIRT-2 +p65+CBP compared to si-Ctrl+p65+CBP. Together, our data suggests that SIRT2 inhibition or deficiency is associated with increased ACp65 expression.

### SIRT-2 inhibition in *ob/ob* septic mice reverses microvascular hypo-inflammation *in vivo*

**Fig 5:**
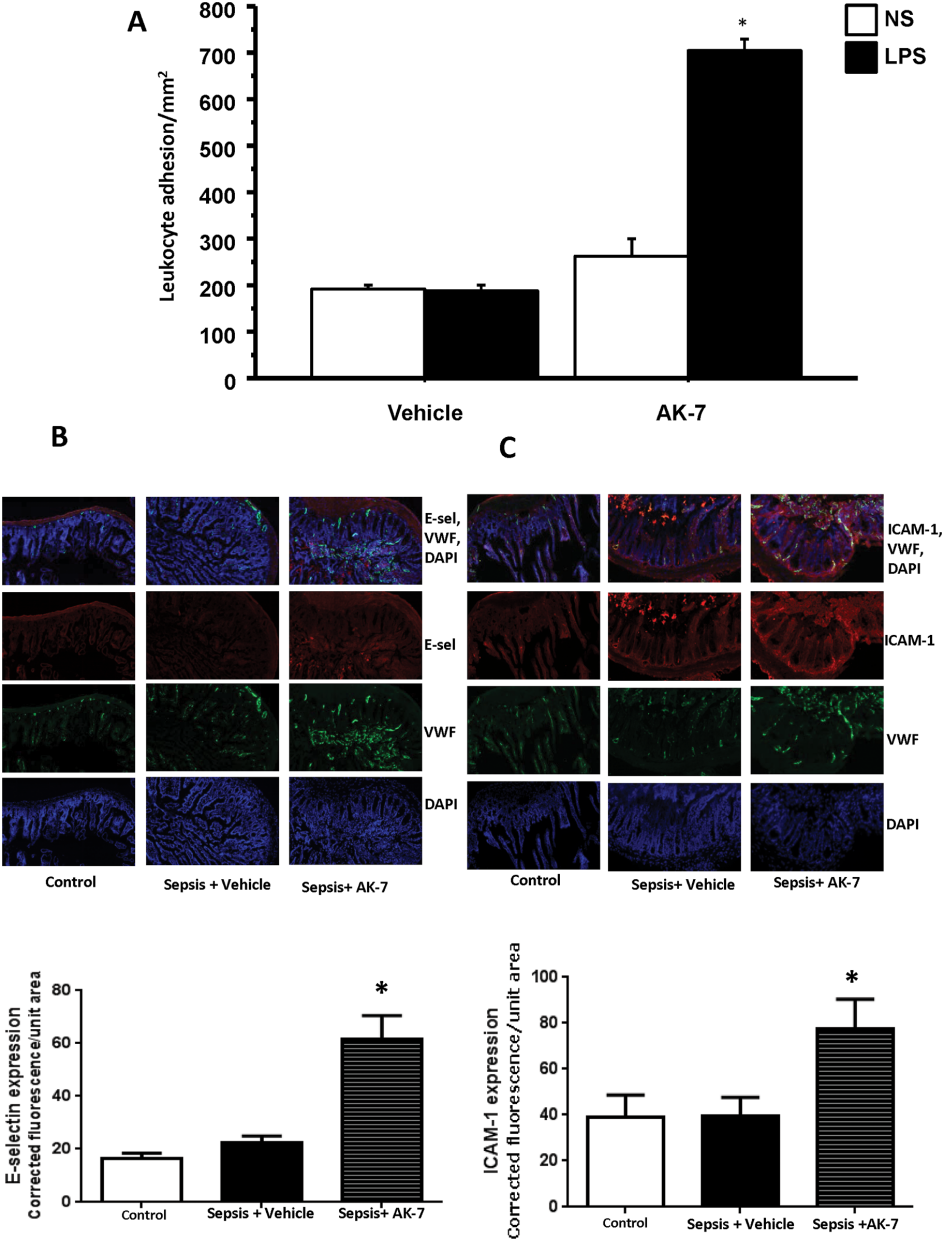
SIRT-2 inhibition in *ob/ob* septic mice restores repressed microvascular leukocyte adhesion. A: SIRT-2 inhibition during hypo-inflammation enhances leukocyte adhesion in *ob/ob*: mice with sepsis: *ob/ob*-septic mice were treated with Vehicle or AK-7 during hypo-inflammatory phase of sepsis, challenged with either normal saline (NS) or LPS and studied leukocyte adhesion 4h later. While Vehicle treated mice showed no further increase in leukocyte adhesion in response to LPS, AK-7 treated mice showed significant increase in leukocyte adhesion in small intestinal microcirculation. * p<0.05 vs. Vehicle +LPS using Tukey‘s post-hoc analysis; error bars: s.e.m. B and C: SIRT-2 inhibition during hypo-inflammatory phase activates endothelium: We treated *ob/ob* mice with either Vehicle of AK-7 during hypo-inflammatory phase of sepsis and studied small intestinal tissue expression of E-selectin and ICAM-1 expression. We co-stained these tissue sections with wither von Willebrand factor or nuclear stain DAPI. We quantified the signal using NIH Image J. There was a significant increase in E-selectin (Fig 5B) and ICAM-1 (Fig 5C) expression in small intestinal tissue sections of Sepsis + AK-7 treated group compared to Sepsis + Vehicle group. * p<0.05 vs. Sepsis + Vehicle using Tukey‘s post-hoc analysis; error bars: s.e.m.

Data in Fig 1A suggests that ob/ob mice show a prolonged hypo-inflammatory phase; AK-7 treatment during the hypo-inflammatory phase of sepsis significantly improves survival. So next, we sought to study whether this beneficial effect on survival is via reversal of hypo-inflammatory phase of sepsis in *ob/ob* mice *in vivo*. Specifically, using endotoxin tolerance *in vivo* as a tool for tracking repressed immunity in the microvasculature, we tested the effect AK-7 in *ob/ob* mice. As shown in Table 3, there were no significant differences in body weight and MAP between different groups studied. We then treated *ob/ob* mice with AK-7/ Vehicle after hypo-inflammatory phase onset (18h post-CLP) and assessed microvascular leukocyte adhesion in response to second-hit LPS. As shown in Fig 5A, vehicle treated mice remain endotoxin tolerant, with no significant difference between Sepsis+NS vs. Sepsis+LPS. However, in AK-7 treated mice, we observed significant increases in leukocyte adhesion in Sepsis +LPS vs. Sepsis +NS groups, indicating reversal of the hypo-inflammatory phase; in contrast with prolonged hypo-inflammation in untreated *ob/ob* mice shown in Fig 1A. Thus, SIRT-2 inhibition with AK-7 reverses hypo-inflammation and restores the microvascular function in *ob/ob*-sepsis mice.

**Table 3:** Body weight and mean arterial blood pressure

	Body Weight (gms) Mean± SEM		MAP (mmHg) Mean± SEM	
	NS	LPS	NS	LPS
Ob/ob sepsis+ Vehicle	37.03±3.9	39.25±1.47	59.0±2.6	60.0±3.08
Ob/ob sepsis+ AK-7	36.34±1.13	39.39±0.41	57.80±5.03	60.40±4.40
DIO sepsis+ Vehicle	33.89±1.03	35.54±2.46	68.6±2.1	58.20±0.73
DIO sepsis+ AK-7	33.89±0.98	38.04±1.13	60.0±4.02	50±5.2

Body weight and mean arterial pressure in *ob/ob* and DIO mice with sepsis with vehicle vs. AK-7 treatment: Body weight and carotid artery mean arterial blood pressure were measured in ob/ob and DIO mice treated with vehicle vs. AK-7 and re-stimulated with normal saline (NS) and LPS. Different groups studied show no significant differences from each other. Similarly, the mean arterial blood pressures (MAP) between different groups were not significantly different from each other.

### AK-7 treatment restores adhesion molecule expression

Having found reactivation of NFkB p65 function after AK-7 treatment in our cells, we tested the effect of SIRT-2 inhibition on p65-dependent adhesion molecules. Rolling and adhesion of circulating leukocytes in microcirculation are facilitated via selectins and ICAM-1, respectively [21,27]. We reported in septic mice that SIRT-1 represses leukocyte adhesion *in vivo* microcirculation, via E-selectin and ICAM-1 expression in lean mice [2,20]. Here, we examined the effect of AK-7 on E-selectin and ICAM-1 expression in the small intestinal microcirculation using immunohistochemistry. As shown in Fig 5B and 5C, E-selectin and ICAM-1 expression increased in AK-7 vs. vehicle treated mice, supporting a role for SIRT-2 in repressing inflammatory reactions in microvasculature during obesity with sepsis.

**Fig 6:**
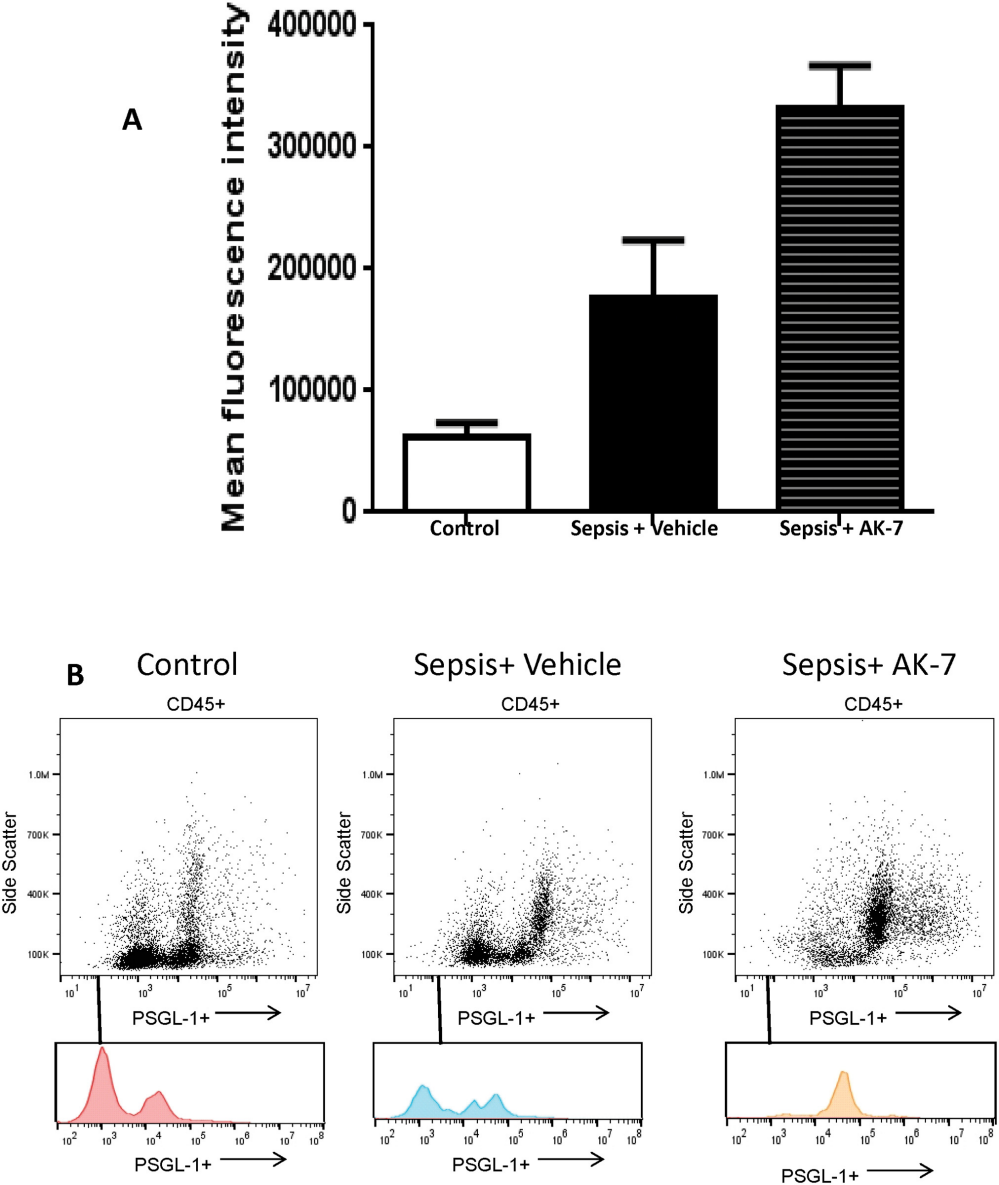
SIRT-2 inhibition during hypo-inflammatory phase activates circulating leukocytes. We treated *ob/ob* mice with either Vehicle of AK-7 during hypo-inflammatory phase of sepsis and studied whole blood leukocytes for PSGL-1 and CD45 expression using flow cytometry. Fig 6A shows mean fluorescence intensity from three different groups while Fig 6B and 6C show representative histogram and dot plots respectively. There was a significant increase in PSGL-1 expression in CD45 positive cells in Sepsis+ AK-7 compared to Sepsis +Vehicle group using Tukey‘s post-hoc analysis; error bars: s.e.m.

AK-7 treatment activates circulating leukocytes: Next, we examined the effect of AK-7 treatment on circulating cells. The circulating leukocytes interact with the selectins expressed on the endothelial cells via P-selectin glycoprotein ligand (PSGL-1), a ligand for E-selectin. We co-stained leukocytes with anti-CD45 antibody and PSGL-1 antibody and gated for CD45 positive cells. As shown in Fig 6, PSGL-1 expression increased in leukocytes of mice treated with AK-7 compared to Vehicle treatment. Fig 6A shows quantification of mean fluorescence intensity CD45+PSGL-1+ cells expression in three groups of mice: Control, Sepsis+ Vehicle and Sepsis+AK-7 treatment. Fig 6B shows representative dot-plots and corresponding histograms for all CD-45+ cells. Together, these data indicate that AK-7 acts both on circulating leukocytes and endothelium during sepsis in *ob/ob* mice.

### Diet induced obese mice with sepsis and SIRT2 expression

**Fig 7:**
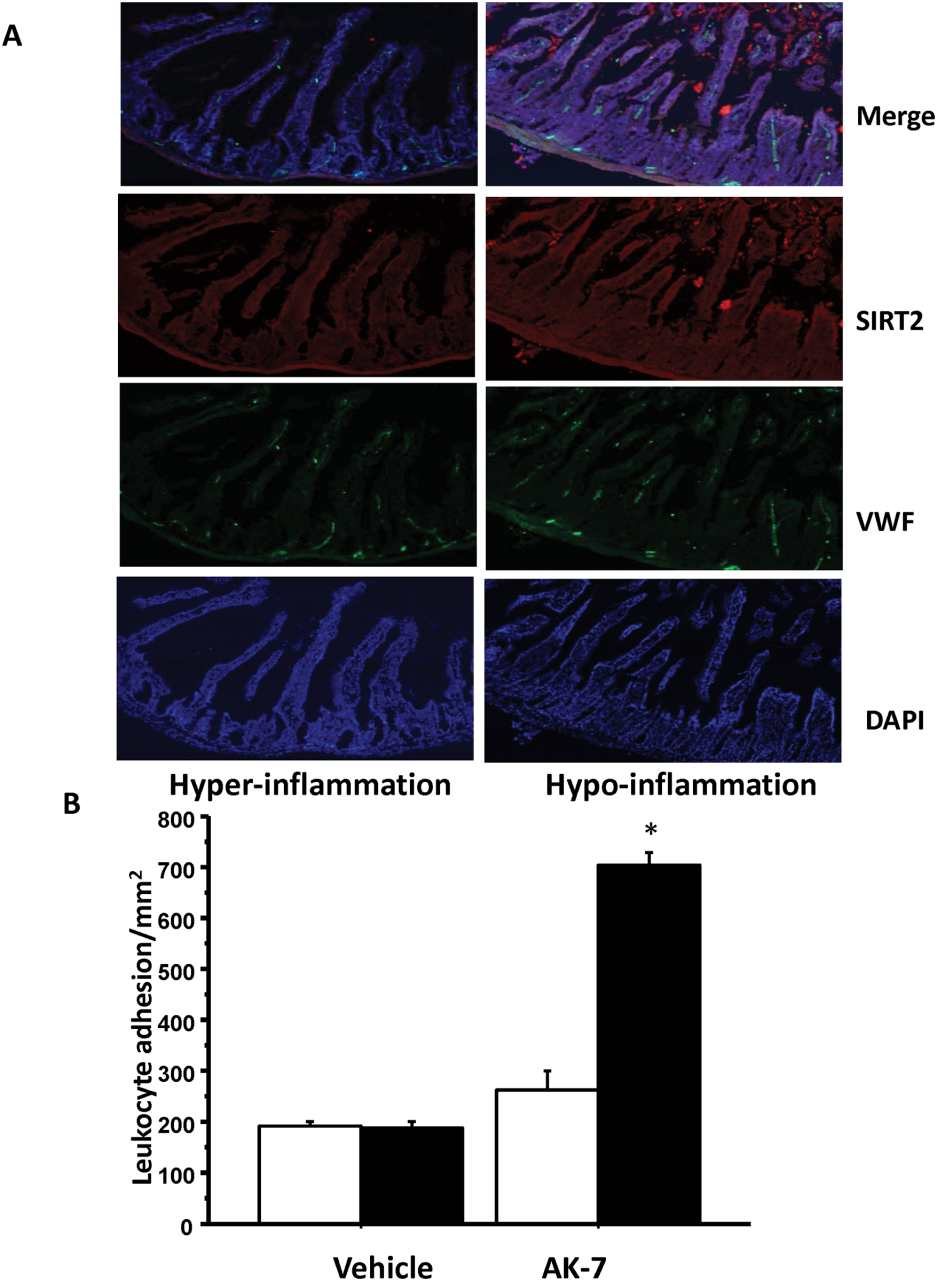
Diet induced obese mice with sepsis and SIRT2 expression. A: Immunohistochemistry of small intestinal tissue (6B) from DIO mice with sepsis were stained for SIRT-2 (Cy3: red), Von Willebrand factor (VWF, FITC: green), nuclear stain (DAPI: blue) and merged color image show that SIRT-2 expression increased during immune repressor/ hypo-inflammatory phase (24 h) Sepsis compared to hyper-inflammatory phase (6 h) similar to that in *ob/ob* mice. B: SIRT-2 inhibition during hypo-inflammation enhances leukocyte adhesion in DIO mice with sepsis. DIO mice with sepsis were treated with Vehicle or AK-7 during hypo-inflammatory phase of sepsis (18 hours post-sepsis), challenged with either normal saline (NS) or LPS and studied leukocyte adhesion 4h later. While Vehicle treated mice showed no further increase in leukocyte adhesion in response to LPS, AK-7 treated mice showed significant increase in leukocyte adhesion in small intestinal microcirculation. * p<0.05 vs. Vehicle +LPS using Tukey‘s post-hoc analysis; error bars: s.e.m.

Finally, we confirmed our key findings in *ob/ob* mice in DIO mice with sepsis. The *ob/ob* mice are leptin deficient and the effect of leptin on innate immunity is controversial [28,29]. We have shown previously that the microvascular inflammation in early sepsis in leptin deficient *ob/ob*, leptin resistant db/db and melanocortin 4 receptor knock out mice associated obese phenotypes are similar[21,30]. We confirmed the key findings of this project in another model of obesity, namely the diet induced obesity (DIO). First we studied whether the hypo-inflammatory phase of sepsis in DIO mice with sepsis is associated with increased SIRT2 expression. As depicted in Fig 7A, there was a marked increase in the SIRT-2 expression small intestinal tissue in DIO mice with sepsis during the hypo-inflammatory phase of sepsis. We also found similar results in liver tissue (data not shown). We then studied microvascular endotoxin tolerance using leukocyte adhesion in the small intestinal microcirculation with and without AK-7 treatment in DIO mice. As shown in Table 3 there were no significant differences in body weight and MAP between different groups. Fig 7B shows that while the vehicle treated DIO sepsis mice remained endotoxin tolerant during the hypo-inflammatory phase, the AK-7 treated mice showed endotoxin responsiveness similar to *ob/ob* mice. Thus, our data suggest that the hypo-inflammatory phase of sepsis is associated with increased SIRT2 expression in DIO mice as well. Moreover, SIRT-2 inhibition during the hypo-inflammatory phase in DIO mice with sepsis reverses the endotoxin tolerance similar to *ob/ob* mice.

## Discussion

The results of this study introduce previously unreported discoveries with potential implications for obesity and sepsis. Specifically, we report for the first time to our knowledge, that SIRT-2 modulates sepsis-related inflammation in obese -septic mice. From the pathophysiologic standpoint, we find that obese septic mice not only increase the magnitude of the initial sepsis inflammatory response in microvasculature, as assessed *in vivo*, but also more rapidly transition in the repressed state of established sepsis, which correlates with profound immune repression of both innate and adaptive immunity[4,31]. Moreover, we discover that the inflammation and immunity repressor hypo-inflammatory phase persists in *ob/ob* septic mice at a lower dose (intensity) of sepsis for at least 7 days, which is significantly longer than we found lean septic mice with equivalent dose of sepsis[2]. Mechanistically, we find that *in vivo* repression of leukocyte adhesion by SIRT-2 is mediated, at least in part, via repression of endothelium (E-selectin, ICAM -1 expression) and circulating cells (PSGL-1 expression); AK-7 treatment reverses both (Figs 5 and 6). We extended mechanistic study in a mouse macrophage cells model *in vitro*, which simulated our *in vivo* results with SIRT-2. SIRT-2 deacetylates master immune and inflammation regulator NFkB p65 thus inactivating p65 function. Taken together, this study implicates NAD+ dependent SIRT-2 deacetylase function as promoting the immune repressive hypo-inflammatory phase and influencing survival in sepsis with obesity. *Ob/ob* mice are leptin deficient. Although the evidence regarding the effect of leptin on inflammatory response is controversial, we have confirmed our findings in other models of obesity such as *db/db* and melanocortin 4 receptor knock out mice[21,30]. In the current project, we confirmed the key findings in clinically significant obesity model, the DIO mice with sepsis. We show that similar to *ob/ob* mice, the hypo-inflammatory phase of DIO mice with sepsis is also associated with increased SIRT-2 expression and SIRT-2 inhibition during the hypo-inflammatory phase reverses it.

Sepsis is the 11th leading cause of death in the US [32], and an even more dominant cause of death worldwide. Sepsis is considered to be the “most expensive condition” in the US[33]. Early, hyper-inflammatory phase of sepsis transitions to a muted inflammatory phase within hours. If homeostasis is quickly retrieved, immune competence and general homeostasis returns, and patient survival is high. If repressed immunity persists, both the original and acquired infections, including latent viruses, are a source of high mortality [3]. Additional mortality is associated with sustained failure of sepsis target organs: heart, lung, kidney, brain, and liver. Mortality remains high in late phase sepsis and has not mechanism-specific treatment. Since the incidence of sepsis is rising, a public health crisis has emerged [34]. Added to this is the epidemic of obesity, which in itself has high morbidities and mortality rates. Sepsis impacts obesity morbidity and mortality.

Endotoxin tolerance is described in sepsis and other systemic inflammatory states[35–37]. Majority of the studies regarding endotoxin tolerance were performed *in vitro*. We adapted this tool to our *in vivo* system to study sepsis-induced endotoxin tolerance in the microvasculature[2]. In the current project we further applied the tool of endotoxin tolerance to assess immune function *in vivo* to obese mice.

Potentially important observation in obesity is that sirtuins are essential sensors and regulators of metabolism [13]. SIRT-1, by far the most studied sirtuin, mobilizes fatty acid and increase gluconeogenesis in liver in addition to improving insulin secretion from pancreas [38]. Emerging data indicate that SIRT-2 also regulates the beta oxidation of fatty acids via PGC1-α pathway [39]. Thus, sirtuins likely play potentially important but so far unknown roles in obesity metabolic and inflammatory pathways. For example, it is known that obesity-associated chronic inflammation—a systemic metabolic syndrome—is associated with a “low SIRT state” [38], which may directly contribute to metabolic syndrome and/or prime obese immune cells for amplified inflammation. This “priming or sensitizing” property may prompt excessive inflammation during early sepsis. Supporting this concept is that SIRT-1 and 2 activation attenuates obesity-related inflammation [39,40].

We have implicated sirtuins as major contributors to sepsis outcome, both early and late [13,41]. Importantly, sirtuins occupy fundamentally important checkpoints for guarding immuno-metabolic homeostasis. Homeostasis deviation is extreme during sepsis. As a rheostat, low expression of SIRT-1 and other sirtuins amplify early sepsis and high expression sustain repressed immunity [20,42]. Immune cells require activation of glycolysis for energy to generate an effector antimicrobial response and promote anabolic channels for growth and differentiation [43]. During the switch of sepsis from glycolysis dependency for energy of hyper-inflammation, immune cells require fatty acid oxidation to supply ATP in a low energy catabolic hypo-inflammatory phase that characterizes repressor immune cells and possibly poor retrieval of organ function [44]. This hypo-inflammatory of sepsis is promoted by SIRT expression and its interaction with AMP kinases [45]. These SIRT- regulated immuno-metabolic processes likely impact many obesity interactions with sepsis.

In this study, we show that at two different doses of sepsis, *ob/ob* mice show decreased survival (S1A and S1B Fig); at lower dose of CLP also show increased immune dysfunction (prolonged hypo-inflammation) compared to lean mice with equivalent dose of CLP. The “survival” observation is in contrast to the clinical observation with controversial data regarding obesity and sepsis-mortality[46]. However, the literature almost unanimously indicates that there is increased morbidity in obese-sepsis vs. lean-sepsis patients[12]. Our data regarding the prolonged hypo-inflammatory immune repressor phase in *ob/ob* mice is consistent with that observation.

Surprisingly, in this study, we found that phenotypic shift from hyper-inflammatory to hypo-inflammatory phase is not controlled by SIRT-1, but rather by SIRT-2; thus, lean and *ob/ob* mice differ in that respect too. SIRT-2 is the most abundant of sirtuins in the white adipose tissue where it critically regulates adipocyte growth in obesity; SIRT2 expression is down- regulated in differentiating pre-adipocytes to increase glycolysis and decrease fatty acid oxidation, allowing adipogenesis to occur. [39,47]. Yet SIRT2 is dominant in mature adipose tissue, perhaps as a checkpoint. Whether SIRT-2 participates in obesity related acute inflammation is largely unknown. Research suggests that while predominantly cytosolic, under acute inflammatory stress, SIRT-2 translocates to the nucleus [48]. This supports our observation that SIRT-2 controls NFkB dependent adhesion molecule gene expression and shifts in NFkB p65 acetylation/deacetylation [49].

This study poses major unanswered questions. First, do the changes of *ob/ob* mice reflect those in nutritional obesity-associated sepsis? From the SIRT perspective this seems likely, but whether SIRT-2 is specific for obesity is unknown. From our data shown in Fig 7 starts to address this important issue. Here we show that the DIO mice also show increased SIRT-2 expression during the hypo-inflammatory phase and SIRT-2 inhibition during the hypo-inflammatory phase reverses it. Secondly, the exact mechanism of how obesity modulates SIRT-2, especially during the acute inflammatory states, needs to be further elucidated. This highly significant concept urges further research in cell, mouse, and human models of obesity.

In summary, we report for the first time to our knowledge, that septic *ob/ob* mice show a prolonged the immune-repressor/ hypo-inflammatory phase that frequently accompanies lethal human sepsis. We implicate SIRT-2 as a novel checkpoint for guarding homeostasis in obesity, but with persistent activity adversely affect survival.

## Supporting Information

S1 Fig Sepsis dose titration for ob/ob mice. We examined 7-day survival in WT lean vs. ob/ob mice. As shown in S1A Fig, the 7-day survival in ob/ob mice with CLP22.2 was significantly decreased (0%) vs. WT (40%) mice. Moreover, all the mice from ob/ob groups died within 48 hours post-sepsis. As shown in S1B Fig, with CLP25.2, the 7-day survival in ob/ob mice was 30% vs. WT mice 100%.* p<0.05 vs. corresponding WT CLP using Log-Rank test.
(TIF)

S2 Fig phases of sepsis in WT mice with CLP25.2. A: In WT mice with CLP25.2, leukocyte adhesion in small intestinal microcirculation (mice n = 5/group) in WT mice with LPS “second-hit” was significantly increased vs. NS groups at 6, 12, 24 and 72 hours post CLP25.2 indicating no hypo-inflammatory phase with CLP25.2 in WT mice. * p<0.05 vs. respective Sepsis+NS group Tukey‘s post-hoc analysis; error bars: s.e.m. B: SIRT2 expression in WT mice with CLP25.2: Small intestinal tissue were stained for SIRT-2 at 6 and 24 hours post CLP25.2. SIRT-2 (Cy3: red), Von Willebrand factor (VWF, FITC: green), nuclear stain (DAPI: blue) and merged color image show that SIRT-2 expression in WT mice at 6 vs. 24 hours post-CLP25.2 remained unchanged.
(TIFF)
