# Correction: Sirtuin-2 Regulates Sepsis Inflammation in *ob/ob* Mice

**DOI:** 10.1371/journal.pone.0162560

**Published:** 2016-09-01

**Authors:** Xianfeng Wang, Nancy L. Buechler, Ayana Martin, Jonathan Wells, Barbara Yoza, Charles E. McCall, Vidula Vachharajani

Bar graphs are missing from Fig 3A of the published article. Please see the correct Fig 3 here.

**Fig 3:**
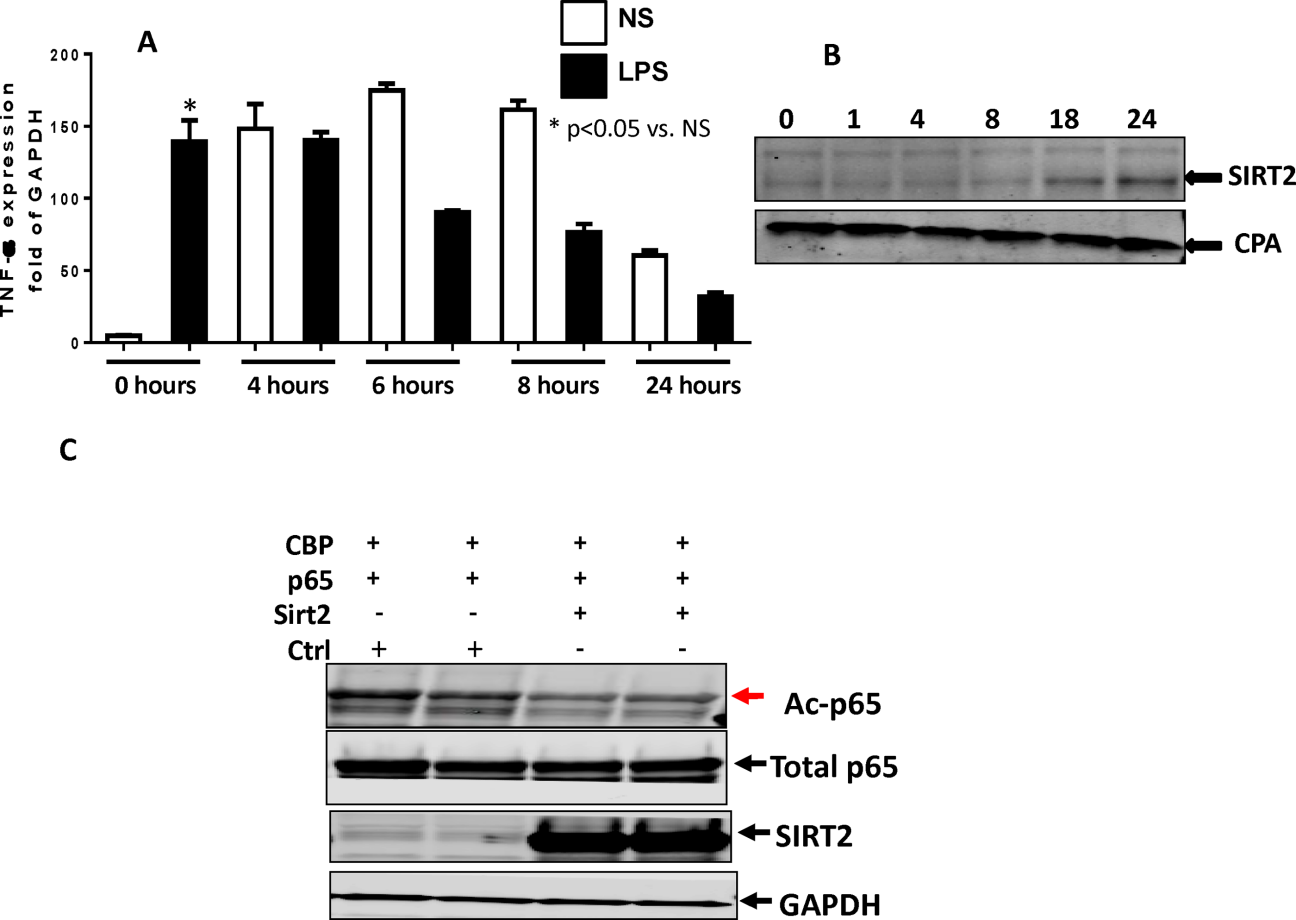
Role of SIRT-2 in the endotoxin tolerant RAW cells. A: RAW cells undergo endotoxin tolerance as early as 4h after LPS stimulation: To study endotoxin tolerance in RAW cells, we stimulated cells with LPS (100ng/ml) and re-stimulated with another LPS challenge (100ng/ml) at 0, 4, 6, 8, and 24 hour time points for four hour. We studied TNF-α mRNA expression. The cells increased TNF-α mRNA expression in response to LPS re-stimulation only at 0h time point. RAW cells were unable to increase TNF-α mRNA further to LPS re-stimulation at 4, 6, 8 and 24h time points, indicating endotoxin tolerance. * p<0.05 vs. respective NS group Tukey‘s post-hoc analysis; error bars: s.e.m. B: SIRT-2 protein expression increased during endotoxin tolerant phase in RAW cells: RAW cells were treated with LPS (100ng/ml) for 0, 1, 4, 8, 18, and 24 h. Whole cell lysates were collected for western blotting of proteins SIRT-2 and CPA (housekeeping gene). Representative image out of three experiments shows that was increased in SIRT-2 expression in 18 and 24h after LPS stimulation. C SIRT-2 deacetylates NFkB p65: We studied the effect of SIRT-2 expression on NFkB p65 acetylation using HEK293 cells. SIRT-2 plasmid was co-transfected with p65 or/and CBP plasmids into HEK293 cells (to increase baseline p65 acetylation) and blotted for antibodies against Ac-p65, total p65, SIRT-2 and GAPDH. NFkB p65 acetylation (Ac-p65) increased in cells with transfection with p65+CBP while it decreased in cells transfected with p65+CBP+SIRT-2, indicating SIRT-2 directly deacetylates NFkB p65.

## References

[1] Wang X, Buechler NL, Martin A, Wells J, Yoza B, McCall CE, et al (2016) Sirtuin-2 Regulates Sepsis Inflammation in *ob/ob* Mice. PLoS ONE 11(8): e0160431 doi: 10.1371/journal.pone.0160431 27500833 10.1371/journal.pone.0160431 PMC4976857

