# Supplementary figures and images for: Sirtuin-2 Regulates Sepsis Inflammation in ob/ob Mice
Source: PLoS One. 2016 Aug 8;11(8):e0160431. doi: 10.1371/journal.pone.0160431 (PMC4976857; doi:10.1371/journal.pone.0160431)

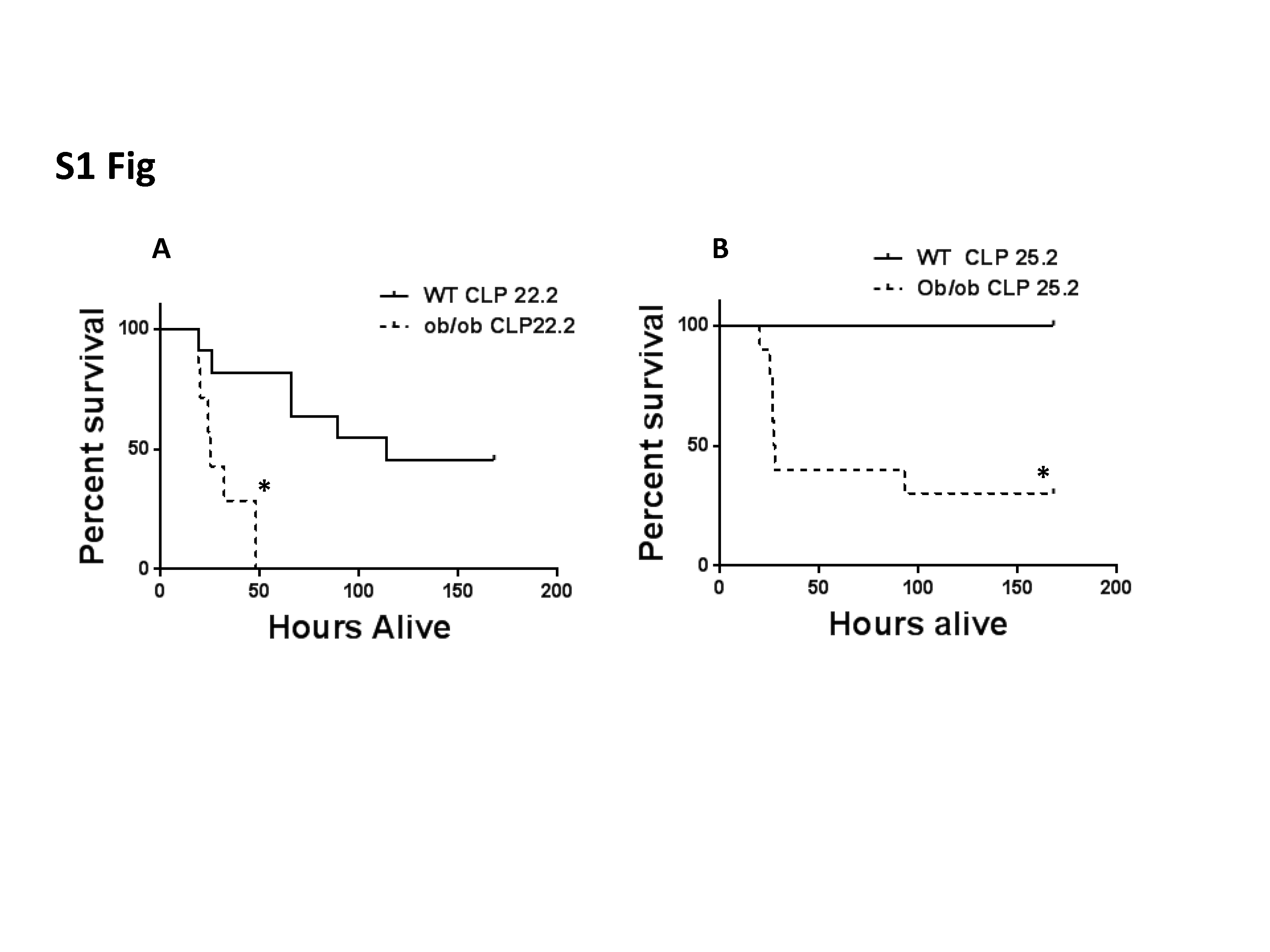

Supplement: S1 Fig — We examined 7-day survival in WT lean vs. ob/ob mice. As shown in S1A Fig, the 7-day survival in ob/ob mice with CLP22.2 was significantly decreased (0%) vs. WT (40%) mice. Moreover, all the mice from ob/ob groups died within 48 hours post-sepsis. As shown in S1B Fig, with CLP25.2, the 7-day survival in ob/ob mice was 30% vs. WT mice 100%.* p<0.05 vs. corresponding WT CLP using Log-Rank test. (TIF) [file pone.0160431.s001.tif]

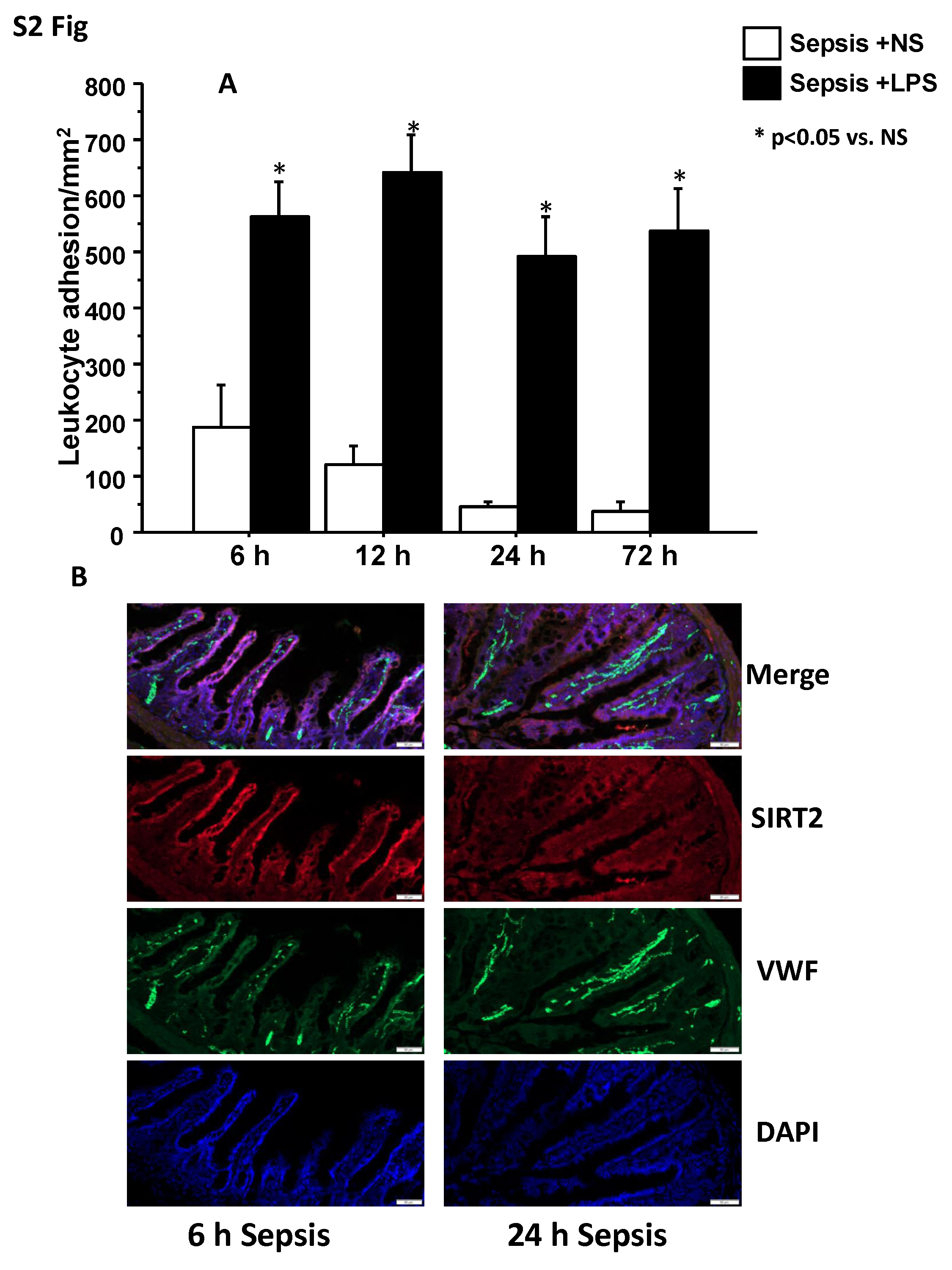

Supplement: S2 Fig — A: In WT mice with CLP25.2, leukocyte adhesion in small intestinal microcirculation (mice n = 5/group) in WT mice with LPS “second-hit” was significantly increased vs. NS groups at 6, 12, 24 and 72 hours post CLP25.2 indicating no hypo-inflammatory phase with CLP25.2 in WT mice. * p<0.05 vs. respective Sepsis+NS group Tukey‘s post-hoc analysis; error bars: s.e.m. B: SIRT2 expression in WT mice with CLP25.2: Small intestinal tissue were stained for SIRT-2 at 6 and 24 hours post CLP25.2. SIRT-2 (Cy3: red), Von Willebrand factor (VWF, FITC: green), nuclear stain (DAPI: blue) and merged color image show that SIRT-2 expression in WT mice at 6 vs. 24 hours post-CLP25.2 remained unchanged. (TIFF) [file pone.0160431.s002.tiff]
